# T‐lymphoblastic transformation of chronic myeloid leukemia

**DOI:** 10.1002/jha2.125

**Published:** 2020-11-06

**Authors:** Maryam Al Bakri, Audi Setiadi

**Affiliations:** ^1^ Department of Pathology and Laboratory Medicine University of British Columbia Vancouver British Columbia Canada

An 85‐year‐old presented with abdominal pain and WBC count of 245.9 × 10^9^/L with 26.9% blasts (66.39 × 10^9^/L): neutrophils 110.66 × 10^9^/L, eosinophils 12.30 × 10^9^/L, basophils 0.0 × 10^9^/L, metamyelocytes 7.38 × 10^9^/L, myelocytes 17.22 × 10^9^/L, lymphocytes 7.38 × 10^9^/L, monocytes 24.59 × 10^9^/L, hemoglobin 97 g/L, and platelets 107 × 10^9^/L. Previous complete blood counts 3 years ago were normal. The blood film showed marked neutrophilia with leukoerythroblastosis, large platelets, and frequent medium‐sized blasts with irregular nuclei (panel A, Wright‐Giemsa stain; original magnification X200 and panel B; original magnification X500). Computed tomography abdomen showed splenomegaly. The patient deteriorated very rapidly and unfortunately passed away before a bone marrow biopsy was obtained. Apart from hydroxyurea, no further treatment was instituted. Flow cytometry of the blood specimen was performed 2 days post‐hydroxyurea, since the pretreatment sample was too old upon receipt at our institution. The flow cytometry result showed two blast populations. The first population (6%) is positive for cCD3, CD5, CD7, CD4, partial CD2, partial CD117, and negative for CD34 and other myeloid/B‐cell antigens (panel C; green population), consistent with T lymphoblasts. There is also a smaller population of blasts (2%) showing myeloid phenotype (panel C; red population), which is favored to be normal left shifted myeloblasts. Cytogenetic analysis demonstrated a complex karyotype with BCR/ABL1 rearrangement in all 10 metaphases examined:: 46,XY,del(8)(q12q21.3),add(9)(q34),del(11)(q23q24), der(14)(14pter → 14q32::?::22q11.2 > 22qter), der(22)t(9;22)(q34;q11.2)[10]. Fluorescence in situ hybridization confirmed BCR/ABL1 fusion in 132/200 nuclei. Given the proportion of cells positive for BCR/ABL1 fusion, myeloid predominance, and smaller fraction of T lymphoblasts, diagnosis of T‐lymphoblastic transformation in the background of chronic myeloid leukemia (CML) is favored rather than de novo T‐cell acute lymphoblastic leukemia (T‐ALL) or mixed phenotype acute leukemia with t(9;22). The latter is much less common and does not usually present with myeloid hyperplasia and left shift, as seen in this case.[Fig jha2125-fig-0001]

**FIGURE 1 jha2125-fig-0001:**
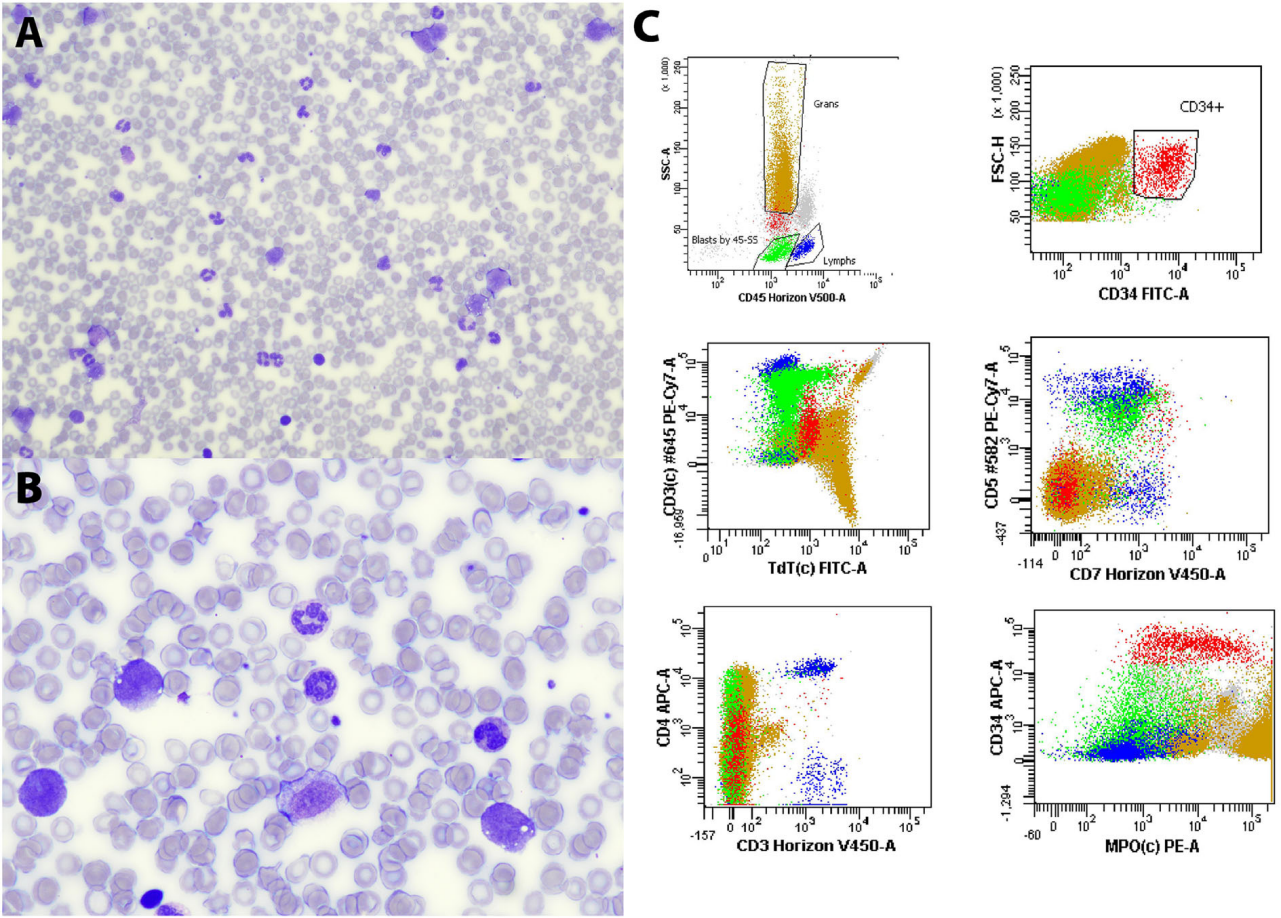
Blood film showing leukoerythroblastosis and medium‐sized blasts (panel A, Wright‐Giemsa stain; original magnification X200 and panel B; original magnification X500). Flow cytometry of peripheral blood showing T‐lymphoblasts (Panel C, green population) and myeloblasts (Panel C, red population). See text for more details.

Lymphoblastic transformation comprises 20‐30% of CML blast phase, the majority being B‐cell lineage. T‐lymphoblastic transformation of CML is very rare with only a few reported cases worldwide and confers a very poor prognosis.^1^ Most cases have a prior history of CML and t(9;22) can be the sole cytogenetic abnormality or as a part of a complex karyotype.^1^ This case is unique since the patient presents with circulating T‐lymphoblasts without a previous history of CML, which poses diagnostic dilemma between de novo T‐ALL, mixed phenotype acute leukemia, or T‐lymphoblastic phase of CML. The diagnosis in this case was even more challenging because patient deteriorated rapidly with no bone marrow biopsy obtained. However, the combination and careful assessment of blood film morphology, flow cytometry, and FISH analysis can aid with the diagnosis of T‐lymphoblastic transformation of CML.

## CONFLICT OF INTEREST

The authors declare no conflict of interest.

## Data Availability

Data sharing not applicable to this article as no datasets were generated or analyzed during the current study.
